# Preclinical pharmacokinetics and metabolism of a novel prototype DNA-PK inhibitor NU7026

**DOI:** 10.1038/sj.bjc.6602823

**Published:** 2005-10-25

**Authors:** B P Nutley, N F Smith, A Hayes, L R Kelland, L Brunton, B T Golding, G C M Smith, N M B Martin, P Workman, F I Raynaud

**Affiliations:** 1 Cancer Research UK Centre for Cancer Therapeutics, The Institute of Cancer Research, Haddow Laboratories, 15 Cotswold Road, Sutton SM2 5NG, UK; 2 Northern Institute of Cancer Research, School of Natural Sciences – Chemistry, Newcastle University, Newcastle upon Tyne NE1 7RU, UK; 3 KuDOS Pharmaceuticals Ltd, 327 Cambridge Science Park, Milton Road, Cambridge CB4 0WG, UK

**Keywords:** DNA-PK, metabolism, pharmacokinetics, NU7026.

## Abstract

In this study we investigated the *in vitro* time dependence of radiosensitisation, pharmacokinetics and metabolism of NU7026, a novel inhibitor of the DNA repair enzyme DNA-dependent protein kinase (DNA-PK). At a dose of 10 *μ*M, which is nontoxic to cells *per se*, a minimum NU7026 exposure of 4 h in combination with 3 Gy radiation is required for a significant radiosensitisation effect in CH1 human ovarian cancer cells. Following intravenous administration to mice at 5 mg kg^−1^, NU7026 underwent rapid plasma clearance (0.108 l h^−1^) and this was largely attributed to extensive metabolism. Bioavailability following interperitoneal (i.p.) and p.o. administration at 20 mg kg^−1^ was 20 and 15%, respectively. Investigation of NU7026 metabolism profiles in plasma and urine indicated that the compound undergoes multiple hydroxylations. A glucuronide conjugate of a bis-hydroxylated metabolite represented the major excretion product in urine. Identification of the major oxidation site as C-2 of the morpholine ring was confirmed by the fact that the plasma clearance of NU7107 (an analogue of NU7026 methylated at C-2 and C-6 of the morpholine ring) was four-fold slower than that of NU7026. The pharmacokinetic simulations performed predict that NU7026 will have to be administered four times per day at 100 mg kg^−1^ i.p. in order to obtain the drug exposure required for radiosensitisation.

There is currently much interest in the discovery and development of novel small molecule inhibitors of enzymes involved in DNA repair (Martin, 2001; Fugmann, 2002). One such enzyme is DNA-dependent protein kinase (DNA-PK), which is a key component of the nonhomologous end-joining pathway of DNA double-strand break (DSB) repair in mammalian cells (Featherstone and Jackson, 1999; Lee and Kim, 2002). DSBs are generated by exogenous insults such as chemical attacks by reactive oxygen species and ionising radiation, the latter remaining an important treatment modality for cancer patients. DNA-PK is a nuclear serine/threonine protein kinase consisting of a heterodimeric DNA-binding subunit (Ku70/80) and a catalytic subunit (DNA-PK_CS_) (Jackson, 1997), and is a member of the phosphoinositide 3-kinase-like kinase (PIKK) family. Mutations in DNA-PK have been identified in radiosensitive cell lines and differential expression has been noted between normal and cancer cell lines (Moll *et al*, 1999; Vaganay-Juery *et al*, 2000; Auckley *et al*, 2001; Eriksson *et al*, 2002). DNA-PK inhibitors, such as LY294002 and wortmannin, and also antisense oligonucleotides have been shown to induce radiosensitisation in a variety of cancer cell lines (Rosenzweig *et al*, 1997; Chernikova *et al*, 1999; Chiosis *et al*, 2001; Kim *et al*, 2002; Sak *et al*, 2002). Furthermore, a deficiency in DNA repair has been reported in the radiosensitive BALB/c mouse (Okayasu *et al*, 2000). Therefore, combining ionising radiation, or any therapy that induces DSBs, with DNA-PK inhibitors may enhance the effectiveness of these therapies (Yarnold, 1997; Yoshida *et al*, 2002). Ideally, the DNA-PK inhibitor should induce radiosensitisation at doses that are nontoxic to nonirradiated cells.

NU7026 (Figure 1) is a novel specific DNA-PK inhibitor (IC_50_: 0.23 *μ*M for DNA-PK, 13 *μ*M for phosphoinositide 3-kinase, >100 *μ*M for ataxia telangiectasia mutated kinase (ATM) and ataxia telangiectasia and rad 3-related kinases (ATR)), which has been shown to sensitise mouse embryonic fibroblasts and Chinese hamster ovary cells to radiation *in vitro* (Veuger *et al*, 2003, 2004; Griffin *et al* 2005). In order to achieve a radiosensitisation effect *in vivo*, the inhibitor should be present in the cells at sufficient concentration and for the duration required to produce radiosensitisation *in vitro* as part of the ongoing optimisation of a chemical series. Of this, NU7026 is a prototype and we have studied the pharmacokinetics and metabolism of this agent in mice following intravenous (i.v.), oral and intraperitoneal (i.p.) administration. In view of possible antitumour experiments combining NU7026 with radiation in animal models, pharmacokinetic modelling of this agent is also presented that suggests a schedule of administration. The importance of metabolism in the elimination of NU7026 is confirmed by studying the pharmacokinetics of a structural analogue in which metabolically sensitive positions on the morpholine ring have been substituted. This study exemplifies some of the challenges faced during the process of lead optimisation. It is often the case that metabolic liability is a major limiting factor in preclinical development. In this specific instance, a dosing regime is suggested that should allow the *in vivo* evaluation of the lead compound. The results also suggest that the structure of NU7026 might be optimised to improve its *in vivo* properties en route to the identification of a compound for clinical administration.

## MATERIALS AND METHODS

### Materials

Unless otherwise stated, materials were from Sigma-Aldrich Company Ltd (Gillingham, Dorset, UK).

### Animals

Female BALB/c mice were supplied by Charles River UK Ltd (Margate, Kent, UK) and maintained on SDA Expanded Rodent diet and water *ad libitum*. All experiments complied with the United Kingdom Coordinating Committee for Cancer Research (UKCCR) guidelines for animal welfare in experimental neoplasia (Workman *et al*, 1997).

### Test compounds

NU7026 (2-(morpholin-4-yl)-benzo[*H*]chromen-4-one), NU7031 (4-(morpholin-4-yl)-6-methoxy-1-benzopyran-2-one) and NU7199 (2-[bis-(2-hydroxyethyl)-amino]-benzo[*H*]chromen-4-one) were synthesised as described (Griffin *et al*, 2005). NU7200 (2-[2-(2-hydroxyethoxy)-ethylamino]-benzo[*H*]chromen-4-one) and NU7107 (2-((2*S*,6*R*)-2,6-dimethylmorpholin-4-yl)-pyrimido[2,1-*a*]isoquinolin-4-one) were both synthesised by a similar procedure to that used for NU7026.

### Radiosensitisation experiments and clonogenic assays

These experiments were not designed as classical radiosensitisation experiments, that is, radiation dose finding study, but rather as experiments to investigate the minimum drug exposure required to observe radiosensitisation. CH1 human ovarian carcinoma cells were chosen as as a model because of their relative radiosensitivity (Kelland, personnal communication). They were grown as monolayers in Dubelcco's modified Eagle's medium (Invitrogen, Paisley, Scotland) augmented with 10% heat-inactivated fetal calf serum, 2 mM L-glutamine, minimal essential medium nonessential amino acid (Invitrogen) and 0.5 *μ*g ml^−1^ hydrocortisone in a 6.5% CO_2_/93.5% air atmosphere.

### Clonogenic survival assay

Cells were seeded at 1000 per 10 ml into 25 cm^3^ flasks and left to attach overnight. Each group consisted of triplicate flasks. Following the addition of NU7026 (10 *μ*M) or dimethyl sulphoxide (DMSO) solvent control for 24 h (final concentration of DMSO was 0.5%), cells were irradiated with a cobalt source (^60^Co) for 3 min and 9 s at a distance of 40 cm to give a total dose of 2 Gy, or 2 min and 49 s at a distance of 30 cm to give a final dose of 3 Gy, or 6 min and 18 s at a distance of 40 cm to give a total dose of 4 Gy. Control cells were not treated with either drug or ionising radiation. Group 2 cells were treated with NU7026 only and Group 3 cells were irradiated only. Groups 4–7 were treated with NU7026 and irradiated with 2, 3 or 4 Gy. The drug was washed off by aspiration of the medium and rinsing with 10 mM phosphate-buffered saline (PBS) 2 h (Group 4), 4 h (Group 5), 6 h (Group 6) and 24 h (Group 7) after exposure. Following the addition of drug-free medium, cells were left to grow until colonies were visible. Colonies were then counted after 9 days and expressed as a percentage of controls. The doubling time of the cells was approximately 16 h.

### *In vivo* experiments

NU7026 was formulated in 10% DMSO and 5% Tween 20 in saline for i.p. and perorally (p.o.) administration at 20 and 50 mg kg^−1^, respectively. For i.v. dosing at 5 mg kg^−1^, NU7026 was formulated in 10% ethanol, 25% PEG 200 and 5% Tween 20 in saline. Control animals received the vehicle alone. Groups of three mice were injected per time point. Blood was collected by cardiac puncture following transient anaesthesia with halothane at 0.083, 0.25, 0.5, 1, 2, 4, 6, and 24 h post administration. Following centrifugation at 1500 *g* for 2 min to obtain plasma, samples were stored at −20°C until analysis. For urinary excretion studies, NU7026 was administered at 5 mg kg^−1^ i.v. Urine was collected over 24 h in metabolic cages, and stored at −20°C until required.

### Analytical method

Samples were analysed by liquid chromatography tandem mass spectrometry (LC/MS/MS). Chromatography was performed using a 50 × 4.6 mm ID 5 *μ*m zwitterionic ABZ+ column (Supelco, Poole, Dorset, UK) and a gradient of 20% methanol (MeOH) in formic acid (0.1%) to 100% MeOH over 3 min, followed by an isocratic period at a flow rate of 0.6 ml min^−1^. The total run time was 7 min. Detection by multiple reaction monitoring was performed on a TSQ700 triple quadrupole mass spectrometer equipped with an electrospray source and operated in positive ion mode (Thermoquest Ltd, Hemel Hempstead, Herts, UK). The heated capillary was maintained at 280°C and at a voltage of 4.5 kV. The following transitions were monitored: m/z (mass to charge ratio) 282.4 → m/z 171 for NU7026; m/z 262.3 → m/z 121 for NU7031; m/z 300 → m/z 256 for NU7119; m/z 300 → m/z 238 for NU7200. Calibration standards (20, 200, 500, 1000, 2000, 5000, 10 000, 20 000 and 50 000 nM) were prepared in blank mouse plasma. Following the addition of internal standard (30 *μ*l of 2 *μ*M solution of NU7031), plasma proteins were precipitated with 300 *μ*l MeOH. Samples were then centrifuged and 20 *μ*l of supernatant was injected onto the column. Peak area was plotted against concentration and unknown concentrations derived from the linear plot (analysed with Prism 2.01, Graphpad Software, San Diego, CA, USA).

For metabolism studies, LC/MS and LC/MS/MS were carried out on an LCQ ion trap instrument (ThermoFinnigan, UK). Separation was achieved using the same column as above. A linear gradient of 90% formic acid (0.1%) : 10% MeOH to 10% formic acid (0.1%) : 90% MeOH was run at 1 ml min^−1^ from 0.5 to 6.5 min followed by 3.5 min isocratic. The capillary temperature was set to 200°C and the scan range from m/z 50 to m/z 850. For confirmation of metabolite structures, metabolites were spiked in control matrix and MS/MS fragmentation patterns and retention times were compared to those of biological samples.

### Pharmacokinetic calculations

Pharmacokinetic parameters were evaluated by noncompartmental analysis (model 200 for p.o. and i.p. administration and 201 for i.v. administration) using WinNonlin® Professional Version 3.2 (Pharsight Corporation, Mountain View, CA, USA). For simulation studies, parameters were derived from compartmental analysis.

## RESULTS

Initially, experiments were carried out to determine the minimum drug exposure required to demonstrate radiosensitisation. Following irradiation of CH1 human ovarian carcinoma cells with 2, 3 and 4 Gy, cell survival was reduced to 51, 28 and 12%, respectively, as measured by a clonogenic survival assay. NU7026 alone (10 *μ*M for 24 h) had minimal effect as shown by a surviving fraction of 91±13% of control cells. However, when combined with radiation, cell survival was reduced to 15, 9 and 1% of the solvent-treated group with 2, 3 and 4 Gy, respectively (Figure 2A). The time course with NU7026 showed that following a 2 h treatment with 10 *μ*M NU7026 in combination with 3 Gy irradiation, there was no significant difference compared to treatment with radiation alone (*t* test, *P*>0.1). However, following a 4, 6 and 24 h exposure to NU7026, a significant radiosensitisation effect was observed (*P*<0.01) with a cell count of 61, 47 and 13% of the radiation control group (Figure 2B). The results show that relatively prolonged exposure of NU7026 is required for radiosensitisation *in vitro*. This suggests that pharmacokinetic properties will be important for the therapeutic activity of this compound.

The analytical method developed for pharmacokinetic studies was specific (no peaks observed in control samples), sensitive (limit of quantitation was 20 nM) and rapid (total run time of 7 min). NU7026 and the internal standard NU7031 were eluted at 7.24 and 6.57 min, respectively. Quality control samples were within 15% of nominal concentrations (data not shown).

The plasma clearance of NU7026 following i.v. administration was rapid and the compound was undetectable at 4 h post administration (Figure 3, Table 1). Compound levels were above 10 *μ*M for less than 1 h. Following i.p. administration at 20 mg kg^−1^, the time course of NU7026 in plasma was very similar to that observed after i.v. administration (Figure 3). Assuming linear pharmacokinetics following i.v. administration, the bioavailability by the i.p. route was 20%. Following oral administration with 50 mg kg^−1^, a maximum concentration of 2.2 *μ*M was observed 1 h after administration. Based on linear i.v. pharmacokinetics, the oral bioavailability was calculated as 15%.

Figure 4A–C show the estimated concentrations of the metabolites based on the relative ion current (signal relative to that of the internal standard) of the metabolites *vs* parent compound in plasma following i.p., i.v. and p.o. administration of NU7026, respectively. This calculation assumes that the metabolites ionise to the same extent as the parent compound. Metabolism was rapid, with peak levels of all metabolites observed 15 min post administration. Assuming that the metabolites and parent compound ionise to the same extent, the major metabolites observed in plasma correspond to m+16 (monohydroxylated product, M1; m/z=298) following administration by all routes. Two metabolites were observed at m+18 (M2 and M3; m/z=300) with M2 showing a higher ion current than M3. In addition, a metabolite M4 was observed at m+48 (m/z=330).

Examination of 24 h urine samples showed trace levels of the parent compound and significant amounts of M1, M2 and M3, but no M4. The most intense signals were observed at m+32 (M5 and M6; m/z=314). In addition, two peaks at m+192 (M7 and M8; m/z=474) were also observed. An m+16 peak (M9) was also detected, but had a different retention time to that of M1 (Figure 5).

**Figure 1:**
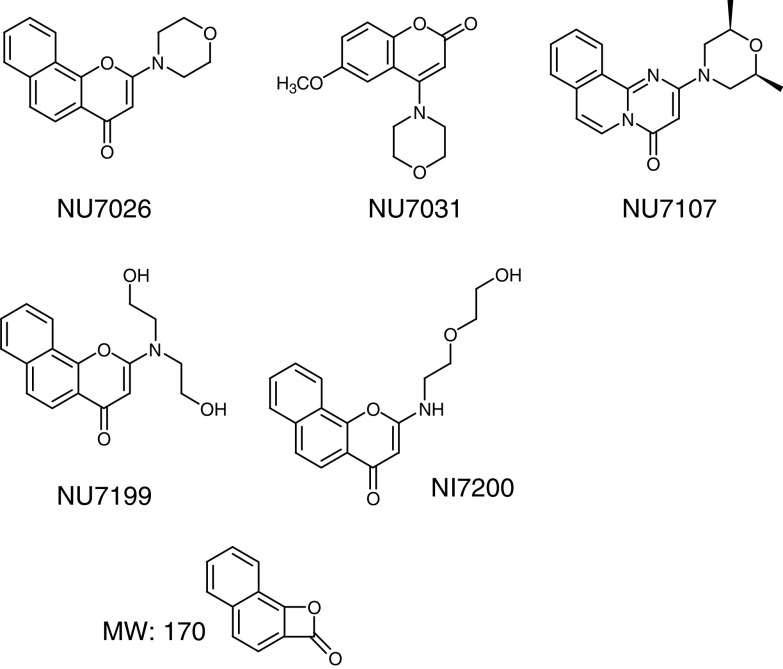
Chemical structures of NU7026, NU7031, NU7107, NU7199, NU7200 and the rearrangement product (naphtopropiolactone) observed in the mass spectrometer (m/z 171) during the fragmentation of NU7026.

**Figure 2:**
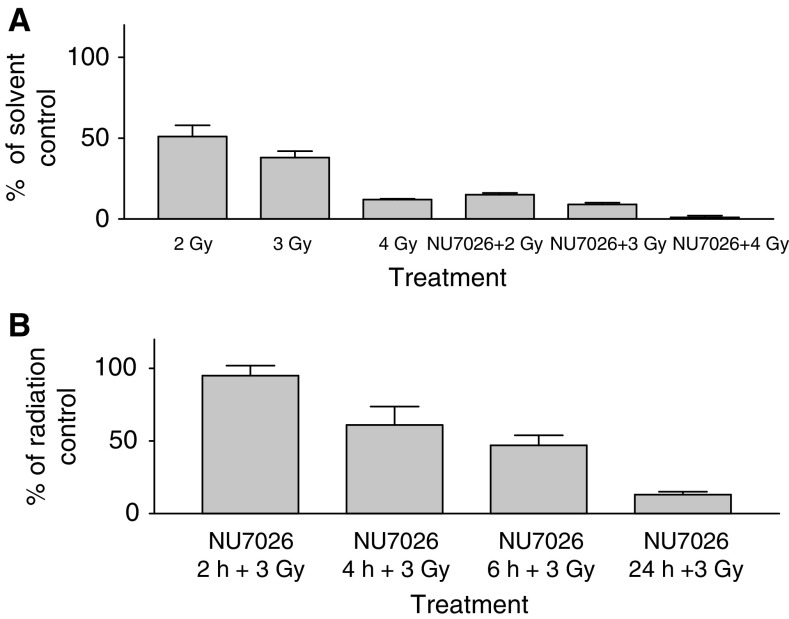
(**A**) Radiosensitisation effect of NU7026 (10 *μ*M) as measured by colony formation assay in radiosensitive CH1 human ovarian cells following 2, 3 and 4 Gy irradiation with a cobalt source. Results are expressed as percentage of control cells (**B**). Time course of the radiosensitisation effect following 10 *μ*M NU7026. Results are expressed as percentage of irradiated controls.

**Figure 3:**
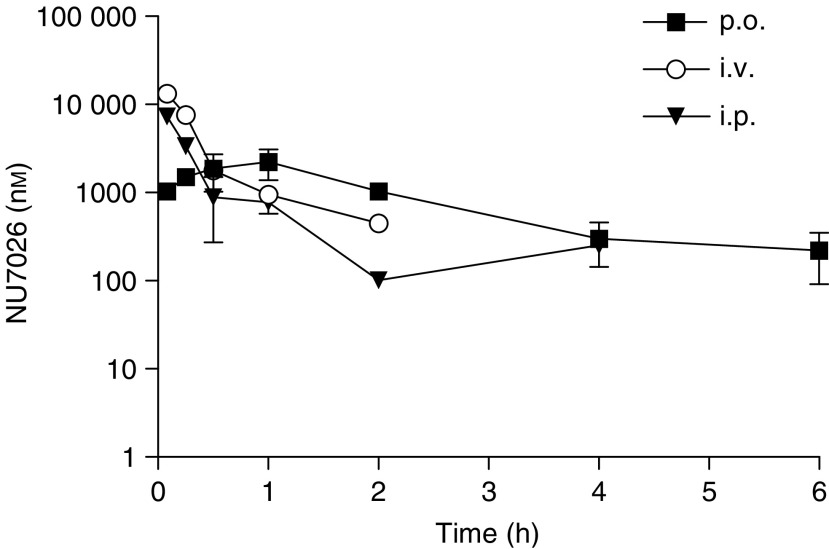
Time dependence of plasma concentration of NU7026 as measured by liquid chromatography tandem mass spectrometry following administration at 5 mg kg^−1^ i.v., 20 mg kg^−1^ i.p. and 50 mg kg^−1^ p.o. to mice. Female BALB/c mice were injected with 0.1 ml/10 g drug solution or vehicle. NU7026 was formulated in 10% DMSO and 5% Tween 20 in saline for i.p. and p.o. administion. For i.v. dosing at 5 mg kg^−1^, NU7026 was formulated in 10% ethanol, 25% PEG 200 and 5% Tween 20 in saline.

**Figure 4:**
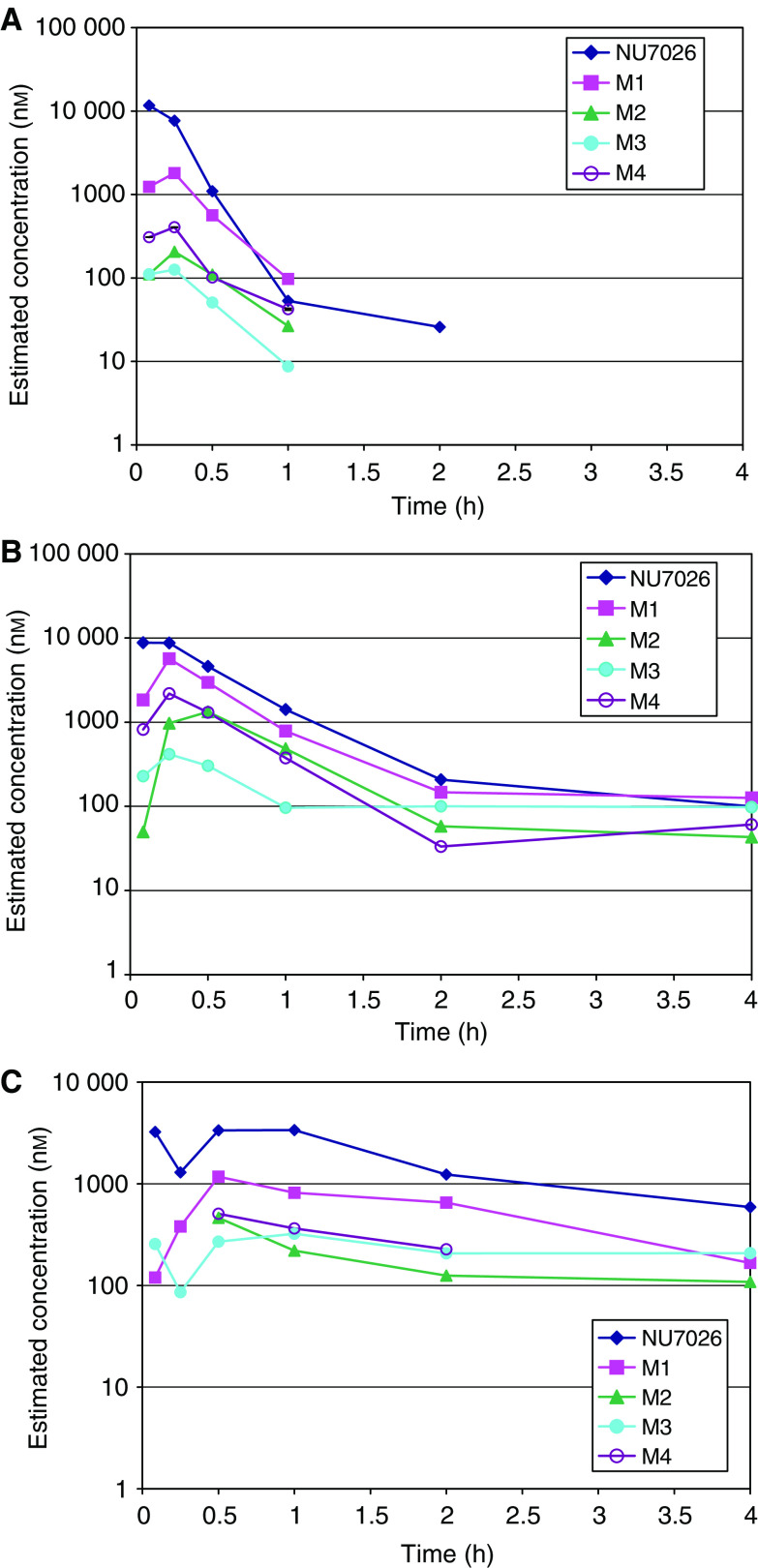
Mouse plasma extrapolated metabolite concentration time profiles measured by total ion current assuming NU7026 metabolites ionise similarly to the parent compound. NU7026 was administered at 5 mg kg^−1^ i.v. (**A**), 20 mg kg^−1^ i.p. (**B**) and 50 mg kg^−1^ p.o. (**C**). NU7026 was formulated in 10% DMSO and 5% Tween 20 in saline for i.p. and p.o. administration at 20 and 50 mg kg^−1^, respectively. For i.v. dosing at 5 mg kg^−1^, NU7026 was formulated in 10% ethanol, 25% PEG 200 and 5% Tween 20 in saline.

**Figure 5:**
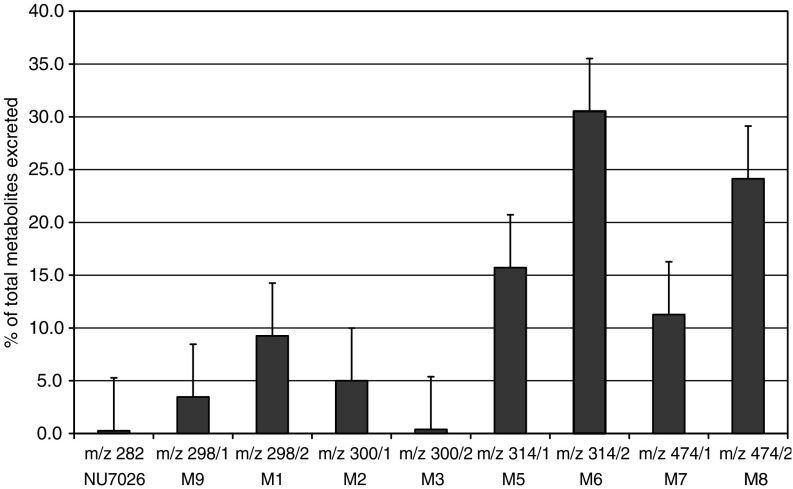
Extrapolated metabolite concentrations measured by LCMS by total ion current in 24 h mouse urine samples following administration of NU7026 at 5 mg kg^−1^ i.v. It is assumed that metabolites and parent compound ionise similarly.

There are several positions on NU7026 that may be susceptible to oxidative metabolism within both the morpholine ring and the benzochromenone ring system. The fragmentation patterns of NU7026 and its plasma metabolites were consistent with the major site of oxidation being at C-2 of the morpholine ring. Fragmentation of the molecular ion (MH^+^) of the parent compound generated a product ion (m/z=171) corresponding to a protonated naphthopropiolactone (Figure 1), which would result from degradation of the pyrone moiety of the benzochromenone system. This fragment was hydroxy-substituted (m/z=187) when hydroxylation occurred on the naphthalene part of the benzochromenone ring system and remained unchanged (m/z=171) when hydroxylation occurred on the morpholine ring. From this observation, together with other fragments generated by the morpholino ring (data not shown), it was deduced that M1 was hydroxylated on the morpholino ring while M9 was hydroxylated on the benzochromenone ring system. M5 and M6 were both bis-hydroxylated on the morpholine ring. Bis-hydroxylation could occur at C-2 and C-6, C-2 and C-3, C-2 and C-5 or C-3 and C-5. For each of these bis-hydroxylation points, there are two possible diastereoisomers. From the information available it is not possible to define the nature of the bis-hydroxylated metabolites, although in each case it is likely that C-2 is hydroxylated, given that oxidation at this position is responsible for the major mono-hydroxylated metabolite (M1). M7 and M8 were identified as glucuronide conjugates of M9 and M1, respectively. M4 corresponded to a tri-hydroxylated product and was a mixture of two compounds, one of which was hydroxylated on the benzochromenone ring system. The fragmentation patterns of M2 and M3 suggested that they were derived from hydroxylation of the morpholine ring at C-2 and C-3, respectively. The products from these hydroxylations (M1 for hydroxylation at C-2) will be in equilibrium with ring-opened aldehyde tautomers, reduction of which affords M2 and M3. In order to confirm the nature of M2 and M3, authentic samples of both NU7199 and NU7200 (Figure 1) were compared to the spectra of the pseudomolecular ions at m/z 300 in plasma and urine. Two peaks were detected at m/z 300 in both plasma and urine and retention times and product ion spectra indicated that M2 corresponds to NU7199 and M3 to NU7200 (data not shown).

**Figure 6:**
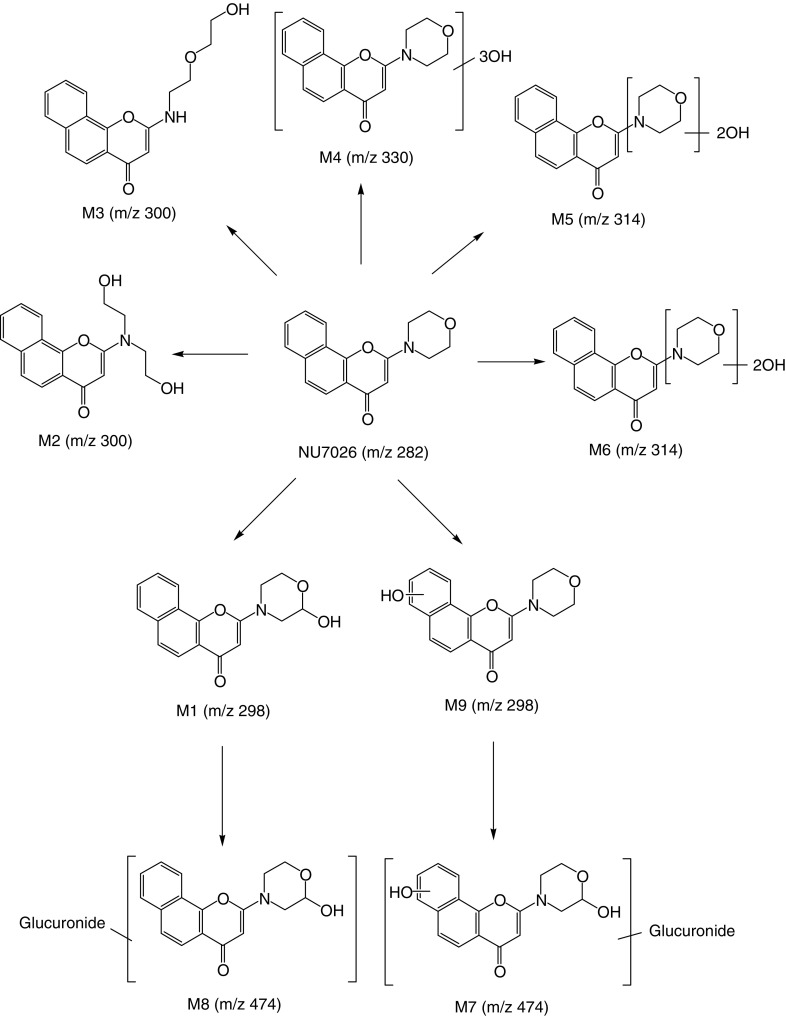
Scheme of the metabolism of NU7026.

Following re-extraction and quantitation (Table 2), it appeared that NU7199 was the most prevalent of the two metabolites (10% in both plasma and urine), thus suggesting that the main hydroxylation position is the 2 position of the morpholino ring. A general scheme of the metabolism of NU7026 is shown in Figure 6.

**Figure 7:**
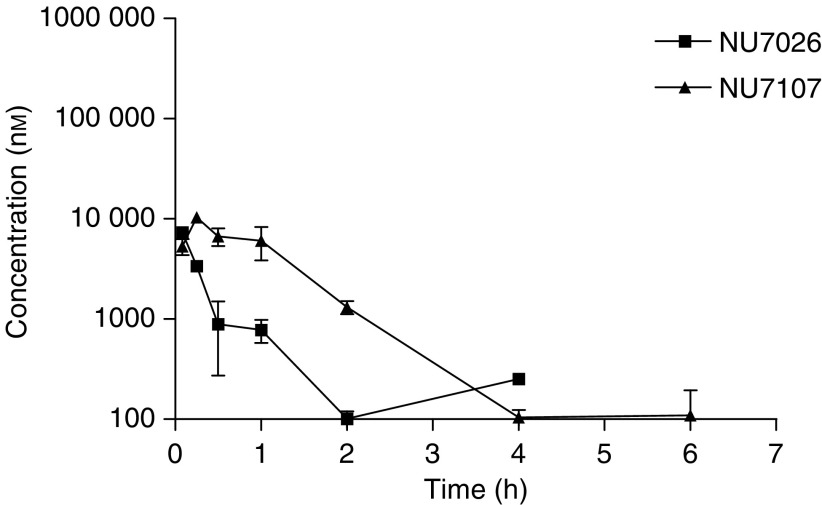
Comparison of plasma concentration *vs* time profiles of NU7026 and NU7107 following i.p. administration at 20 mg kg^−1^ in 10% DMSO to female BALB/c mice. Measurements are done by LC/MS/MS with multiple reaction monitoring following addition of internal standard and plasma extraction by protein precipitation. Quantification was performed by external calibration.

In order to confirm if substitution of the hydroxylation positions could decrease metabolism and therefore plasma clearance, a bis-methylated morpholino derivative of NU7026 (NU7107) (Figure 1) was synthesised and administered to mice i.p. at 20 mg kg^−1^. The pharmacokinetic profile of NU7107 was significantly improved compared with NU7026 (Figure 7, Table 3), with a four-fold decrease in plasma clearance. Although there was no significant difference in the terminal half-life, NU7107 was still detectable 6 h post administration (Figure 7). Although NU7107 is significantly weaker at inhibiting DNA-PK (IC50>10 *μ*M) in comparison to NU7026, synthesis of this compound demonstrated the importance of metabolism in the clearance of NU7026 and showed that pharmacokinetic properties can be modulated by the appropriate substitution.

**Figure 8:**
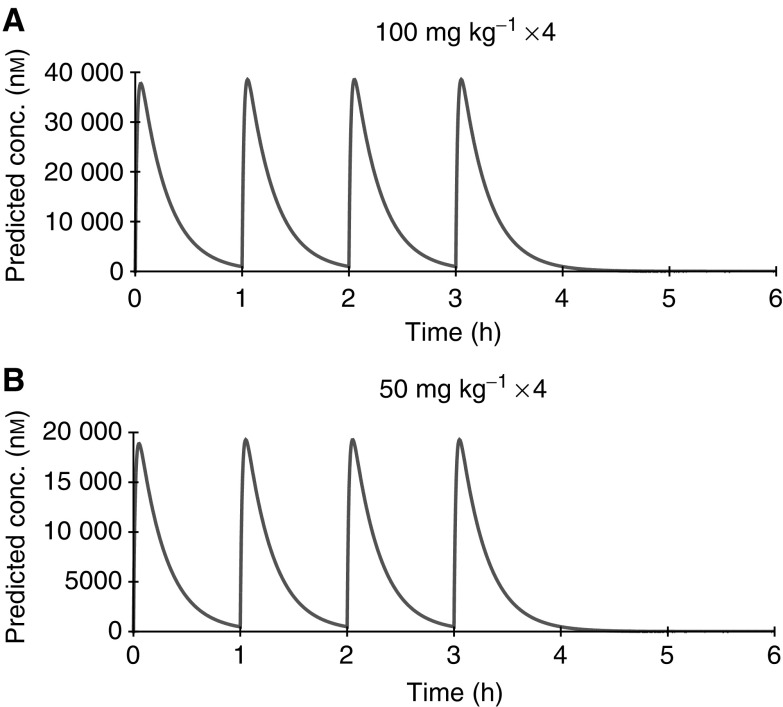
(**A**, **B**) Simulation study of plasma concentrations following 4 h repeat administrations of NU7026 at 100 and 50 mg kg^−1^ i.p. The simulation was carried out using parameters determined by WinNonlin compartmental analysis.

Pharmacokinetic simulations performed for a schedule of administration of NU7026 i.p. are shown in Figure 8. Following four repeat administrations of 50 or 100 mg kg^−1^ NU7026 at 1 h intervals, NU7026 was present at concentrations above 10 *μ*M for approximately 2 and 4 h, respectively.

## DISCUSSION

The current investigation describes the radiosensitisation, pharmacokinetic and metabolism studies of a novel DNA-PK inhibitor NU7026. This compound has similar structural features to LY294002, in particular the morpholino group, but unlike LY294002 has no inhibitory effect on phosphoinositide 3-kinase (Veuger *et al*, 2003, 2004; Griffin *et al*, 2005). A recent report on IC87361 shows a significant radiosensitisation in LCC and B16F0 cells with 6 Gy, which translates into a significant growth delay in xenograft models (Shinohara *et al*, 2005). Our study shows that at 10 *μ*M, the compound alone has no intrinsic growth inhibition properties but it significantly potentiates the effect of radiation in CH1 human ovarian cancer cells. The radiosensitisation effect of this compound has previously been shown to compare very well with results that have been reported for LY294002 and wortmannin (Rosenzweig *et al*, 1997; Fukuchi *et al*, 2000; Kim *et al*, 2002). We examined the effect of drug exposure on the relatively radiosensitive CH1 human ovarian carcinoma cell line. In previous experiments in Chinese hamster ovary cells and mouse embryonic fibroblasts (Veuger *et al*, 2003, 2004), cells were preincubated with the drug for 24 h prior to irradiation. Here, we have evaluated the minimum exposure required to observe a radiosensitisation effect. We found that at least 4 h exposure at 10 *μ*M NU7026 is necessary and that 24 h produced an even greater effect. This requirement may be dependent on the kinetics of cellular uptake of the drug, the *k*_on_ and *k*_off_ of the inhibitor as well as the kinetics of double strand DNA repair. These *in vitro* experiments are important because they suggest that drug exposure is likely to be important *in vivo* and hence that pharmacokinetic properties of the agents will be important for the therapeutic effects of NU7026. The pharmacokinetics of NU7026 show that the drug is cleared very rapidly from the general circulation, with plasma clearance values equivalent to mouse liver blood flow. Assuming that the pharmacokinetics of NU7026 are linear, the i.p. and p.o. bioavailability of this agent are 20 and 15%, respectively. The chief cause for the rapid clearance was oxidative metabolism. Multiple oxidations were identified followed by glucuronidation as the major metabolic routes. Our study clearly indicates that the main site of oxidative metabolism is at C-2 of the morpholino ring. This finding was confirmed by comparison with synthetic reference standards. Furthermore, we showed that NU7107, which has two positions on the morpholino ring blocked for metabolism by methyl groups, has a four-fold lower plasma clearance compared to NU7026.

We have previously evaluated the pharmacokinetics and metabolism of LY294002 and the morpholino position was also found to be oxidised extensively for this compound (Nutley *et al*, 2001). Monohydroxylation occurs mainly on position 2 of the morpholine group, which results in opening of the ring system. Hydroxylation on morpholino rings attached to heterocycles has been reported previously and N-oxidations have also been described (Langner *et al*, 1993; Baker *et al*, 1999; Huskey *et al*, 2004; Zhang *et al*, 2004; Zhao *et al*, 2004). Our study suggests that the modest bioavailability of NU7026 is not due to first pass metabolism as the relative proportion of administered dose present as parent or metabolites are similar after i.v., i.p. and p.o. administration. Further studies are necessary, however, to evaluate the enzymes responsible for these oxidations and for the assessment of tissue concentrations. In any case, the *C*_max_ observed after p.o. administration of 50 mg kg^−1^ (<2500 nM) suggest that 10 *μ*M concentrations cannot be sustained for 4 h after 200 mg kg^−1^ NU7026. An alternative would be to dose by constant infusion with osmotic minipumps, but this is not possible due to insufficient solubility of NU7026.

Based on the experimental results reported here, the pharmacokinetic results suggest that 24 h drug exposure will not be achievable *in vivo.* Pharmacokinetic modelling has shown that a schedule of 100 mg kg^−1^ i.p. administered four times at 1 h intervals should produce the required exposure to NU7026 for radiosensitisation *in vivo*. It will then be interesting to compare the results with other less-specific inhibitors such as LY294002 (Hu *et al*, 2000; Edwards *et al*, 2002; Semba *et al*, 2002; Gupta *et al*, 2003; Shinohara *et al*, 2005). If this dose is not tolerated, then it will be necessary to develop compounds that are either more potent or less readily metabolised. These findings, showing the metabolic hotspots in NU7026 provide a basis for further optimisation in this drug discovery programme.

## Figures and Tables

**Table 1:** Plasma pharmacokinetics following administration of NU7026 to mice at 20 mg kg^−1^ i.p., 5 mg kg^−1^ i.v. and 50 mg kg^−1^ p.o.

**Route**	***C*_max_ (nmol l^−1^)**	***T*_max_ (h)**	**AUC last (nM l h^−1^)**	***t*_1/2_ (h)**	**Cl obs. (l h^−1^)**	***V*_d_ (l)**
I.p.	7359	0.083	2940	0.87	0.44	0.55
I.v.	5123	0	3616	1.22	0.09	0.16
P.o.	2230	1	5191	1.48	0.63	1.34

Parameters are *C*_max_ (maximum concentration), AUC (area under the concentration *vs* time curve), Cl (clearance), *t*_1/2_ (half-life), *V*_d_ (volume of distribution based on the elimination phase).

Parameters were calculated by noncompartmental analysis.

**Table 2:** Plasma pharmacokinetics of NU7026 and its metabolites NU7199 and NU7200 following administration of NU7026 at 5 mg kg^−1^ i.v. to mice

**Compound**	***C*_max_ (nmol l^−1^)**	***T*_max_ (h)**	**AUC last (nmol l h^−1^)**	***t*_1/2_ (h)**	**Cl obs. (l h^−1^)**	***V*_d_ (l)**
7026	5123	0	3616	1.22	0.09	0.16
7199	705	0.25	414	0.2	0.82	0.23
7200	293	0.25	143	0.16	2.44	0.55

Parameters are *C*_max_ (maximum concentration), AUC (area under the concentration *vs* time curve), Cl (Clearance), *t*_1/2_ (half-life), *V*_d_ (volume ofz distribution based on the elimination phase).

**Table 3:** Plasma pharmacokinetics of NU7026 and NU7107 following administration at 20 mg kg^−1^ i.p. to mice

**Compound**	***C*_max_ (nmol l^−1^)**	***T*_max_ (h)**	**AUC last (nmol l h^−1^)**	***t*_1/2_ (h)**	**Cl obs. (l h^−1^)**	***V*_d_ (l)**
7026	5123	0.083	7349	0.87	0.44	0.55
7107	10390	0.25	12165	0.79	0.11	0.12

Parameters are *C*_max_ (maximum concentration), AUC (area under the concentration *vs* time curve), Cl (clearance), *t*_1/2_ (half-life), *V*_d_ (volume of distribution based on the elimination phase).
